# Risk Factor Analysis for Occurrence of Linezolid-Resistant Bacteria in the Digestive and Respiratory Tract of Food-Producing Animals in Belgium: A Pilot Study

**DOI:** 10.3390/antibiotics13080707

**Published:** 2024-07-29

**Authors:** Michèle Driesen, Michaël Timmermans, Mickaël Cargnel, Xavier Simons, Maria-Eleni Filippitzi, Boudewijn Catry, Fabiana Dal Pozzo, Wannes Vanderhaeghen, Bénédicte Callens, Marc Dispas, Cécile Boland

**Affiliations:** 1 Coordination of Veterinary Activities and Veterinary Epidemiology, Department of Infectious Diseases in Animals, Sciensano, 1050 Brussels, Belgium; michele.driesen@sciensano.be (M.D.); mickael.cargnel@sciensano.be (M.C.); xavier.simons@sciensano.be (X.S.); 2 Veterinary Bacteriology, Department of Infectious Diseases in Animals, Sciensano, 1050 Brussels, Belgium; mic_tim@hotmail.com; 3 Laboratory of Animal Health Economics, Aristotle University of Thessaloniki, University Campus, 54124 Thessaloniki, Greece; mefilippi@vet.auth.gr; 4 Healthcare-Associated Infections and Antimicrobial Resistance, Department of Epidemiology and Public Health, Sciensano, 1050 Brussels, Belgium; boudewijn.catry@sciensano.be; 5 Faculty of Medicine, Université Libre de Bruxelles, 1070 Brussels, Belgium; 6 Center of Expertise on Antimicrobial Consumption and Resistance in Animals, 1210 Brussels, Belgium; fabiana.dalpozzo@amcra.be (F.D.P.); wannes.vanderhaeghen@amcra.be (W.V.); benedicte.callens@amcra.be (B.C.); 7 Health Information, Department of Epidemiology and Public Health, Sciensano, 1050 Brussels, Belgium; marc.dispas@sciensano.be

**Keywords:** linezolid resistance, florfenicol use, antimicrobial use, risk factors, One Health, antimicrobial resistance

## Abstract

Linezolid is a critically important antimicrobial used in human medicine. While linezolid is not licensed for food-producing animals, the veterinary use of other antimicrobials, such as phenicols (e.g., florfenicol), could cross/co-select for linezolid-resistant (LR) bacteria. Such LR strains pose a great concern for public health due to their potential transfer between animals and humans. This study explored possible associations between epidemiological risk factors, including phenicol use, and the occurrence of LR bacteria, such as enterococci and staphylococci, in poultry, pigs, and veal calves in Belgium. Florfenicol use significantly increased the likelihood of harboring LR bacteria in veal calves, sows, and fattening pigs, particularly for the digestive tract (odds ratio (OR): [3.19–5.29]) and the respiratory tract (OR: [6.11–9.09]). LR strains from feces from fattening pigs were significantly associated with production type (OR: [3.31–44.14]) and the presence of other animal species (OR: 0.41). The occurrence of LR strains in the respiratory tract from sows was also significantly associated with using antimicrobials other than florfenicol (OR: 10.07) and purchasing animals (OR: 7.28). Our study highlights the potential risks of using certain veterinary antimicrobials, such as florfenicol, in food-producing animals and emphasizes the need for responsible antimicrobial use to safeguard both animal and public health.

## 1. Introduction

Linezolid is a synthetic oxazolidinone antimicrobial with activity against Gram-positive bacteria. It is a critically important antimicrobial [1] in human medicine used to treat highly resistant infections caused by methicillin-resistant *Staphylococcus aureus* (MRSA) and vancomycin-resistant enterococci (VRE). The emergence of linezolid-resistant (LR) strains in food-producing animals in several European countries [2,3] bears a potential risk to public health due to the possible transfer of LR genes between animals and humans, either via the direct contact of humans with living animals (e.g., livestock) or indirectly via the food chain [4]. While linezolid is not licensed for food-producing animals [5], cross-selection with the veterinary use of other antimicrobial agents, such as phenicols, is plausible. As a matter of fact, different reported LR genes (i.e., *optrA*, *poxtA,* and/or *cfr*) in bacteria derived from food-producing animals [3,6,7] also confer reduced susceptibility to phenicols [8,9,10]. While chloramphenicol is no longer allowed in food-producing animals due to food safety concerns (in the European Union, since 1994 [11]), florfenicol is routinely used for a variety of mainly respiratory infections in livestock. The observation that LR genes can co-occur with other phenicol resistance genes (*fexA* or fexB) [3,12] further supports the hypothesis of the co-selection of LR genes by phenicols.

The sales of phenicols in veterinary medicine have increased in Belgium in the last decade: the sold mg per kg of biomass more than doubled between 2012 and 2018 (from 0.71 mg/kg biomass in 2012 to 1.59 mg/kg in 2018 [13]), yet it has stayed relatively stable since [14]. According to the current Belgian guidelines on responsible antibacterial use (published by AMCRA, Belgian Centre of Expertise on AntiMicrobial Consumption and Resistance in Animals), florfenicol is classified within the antibacterial classes as having the lowest importance for human medicine in terms of resistance selection and transfer [15]. In veterinary medicine, in most cases, it is recommended as a first choice of treatment for respiratory infections in cattle and pigs [16,17]. The European Medicines Agency (EMA) places amphenicols (florfenicol and thiamphenicol) in Category C (“Caution”) for use in animals as an antimicrobial class with a high probability of resistance transfer that may lead to resistance to last-resort antimicrobial classes [18]. In human medicine, for antimicrobials in this category, there are, in general, alternatives. However, few or no antimicrobial alternative treatments presenting a lower risk (Category D “Prudence”) are available for some major veterinary indications [18], which may justify the use of this category of antimicrobials in animals.

Prior research on LR has been rather scarce, primarily focusing on the identification and characterization of LR genes in staphylococci and enterococci (e.g., [3,6,7]) as well as their susceptibility to other antimicrobials (phenicols, lincosamides, oxazolidinones, pleuromutilins, streptogramin A and/or tetracycline) (e.g., [8,9,10]). Nonetheless, a significant knowledge gap remains in understanding the risk factors favoring the occurrence and dissemination of these resistance genes in food-producing animals. In the present pilot study, we explored potential associations between phenicol usage and other putative risk factors with the occurrence of LR enterococci, staphylococci, and one *Pediococcus pentosaceus* retrieved in feces from veal calves, broilers, laying hens, and pigs, as well as in nasal samples from pigs. This investigation used data obtained and analyzed during 2019–2020 from samples collected in Belgium in 2019 [3] and national database records, aiming to shed light on this critical issue. Whole-genome sequence data available from a previous study [3] were used to investigate further the potential genetic background of cross/co-selection with other types of antibiotic use that could support the associations found.

## 2. Results

### 2.1. Putative Risk Factors for Linezolid Resistance (LR)

The literature search identified a total of 180 unique publications, of which 22 relevant articles (Table S1 in Supplementary Materials) identified potential risk factors for linezolid resistance. Table 1 shows the final list of putative risk factors for the present study.

### 2.2. Study Population

The number of herds that are included in this study and are grouped within linezolid-resistant positive (LRP) and linezolid-resistant negative (LRN) groups by each of the investigated categorical and continuous variables are described in Table 2 (fecal samples) and Table 3 (nasal swab samples). In total, 27.4% (31/113) of herds with veal calves, 12.6% (31/247) of herds with fattening pigs, 1.7% (3/179) of laying hen flocks and 6.8% (13/190) of broiler flocks were found to be LRP in the feces examinations. In the nasal swab analysis, 24.6% (17/69) of herds with fattening pigs and 20.8% (16/77) of herds with sows were found to be LRP.

### 2.3. Fecal Samples

The results of the univariable analysis that determine the association between putative risk factors and the occurrence of LR bacteria in the feces of veal calves, fattening pigs, laying hens, and broilers are presented in Table 4.

#### 2.3.1. Veal Calves

Univariate logistic regression analysis in feces showed that the florfenicol use during the estimated stay of veal calves on the farm prior to sampling increased the odds of finding LR bacteria by more than three times (OR: 3.19, 95% confidence interval (CI): 1.36–7.97, *p*-value: 0.01) compared to no use. The findings suggest that the use of phenicols increases the likelihood of observing LR strains in veal calves.

#### 2.3.2. Fattening Pigs

Based on the univariate logistic regression analysis, the use of florfenicol increased the odds of finding LR bacteria in feces from fattening pigs by more than five times (OR: 5.29, 95% CI: 2.39–11.78, *p*-value: <0.01). The odds for LR were 3.31 times (OR: 3.31, 95%CI: 1.14–12.90, *p*-value: 0.03) higher among fattening herds and 44.14 times (OR: 44.14, 95%CI: 1.99–6971.80, *p*-value: 0.02) higher among breeding herds and piglet-rearing herds compared with closed herds. The significant results on breeding and piglet-rearing herds should be taken with caution because only one herd of these categories was sampled. The presence of other animal species on the farm (bovine, sheep, goat, poultry) reduced the odds for LR compared to the absence of other animal species by approximately 59% since the odds are reduced by a factor of 0.41 (OR: 0.41, 95%CI: 0.15–0.95, *p*-value: 0.04).

#### 2.3.3. Laying Hens and Broilers

In laying hens and broilers, no significant associations were found in the univariate logistic regression analysis, except for the herd size in broilers (OR of 1.00, 95%CI: 1.00–1.00, *p*-value: 0.03), yet the strength of the association indicated that the odds for LR did not change with herd size when rounded to two decimal places. Interestingly, although there have been no or minimal recent occurrences of phenicol usage in the studied poultry flocks (0.0% of laying hens flocks and 0.5% of broilers flocks with florfenicol use), LR bacteria were found in 1.68% (n = 3/179) of the studied laying hen flocks and 6.84% (n = 13/190) of the studied broiler flocks.

### 2.4. Nasal Swab Samples

The results of the univariable analysis, which determine the association between putative risk factors and the occurrence of LR bacteria in the nasal swabs of fattening pigs and sows, are presented in Table 5.

#### 2.4.1. Fattening Pigs

Univariate logistic regression analysis in nasal swabs showed that the odds for LR bacteria were approximately 9.09 times greater in farms that treated fattening pigs with florfenicol compared with farms that did not (OR: 9.09, 95%CI: 1.92–56.15, *p*-value: 0.01). Note that the large confidence interval may be caused by the small number of resistant herds (n = 5) in the dataset associated with florfenicol use.

#### 2.4.2. Sows

The univariate logistic regression model in sows showed that farms that treated sows with florfenicol had 6.11 times greater odds for LR bacteria compared to farms that did not (OR: 6.11, 95%CI: 1.51–26.25, *p*-value: 0.01). General antimicrobial use (excluding florfenicol use) increased the odds for LR by a factor of 10.07 (OR: 10.07, 95%CI: 1.22–1313.51, *p*-value: 0.03) compared to not using other antimicrobials. Purchasing animals from other farms increased the odds for LR by a factor of 7.28 (OR: 7.28, 95%CI: 2.00–39.26, *p*-value: <0.01) compared to no purchase of animals. 

### 2.5. General Observations among All Samples

No other factors were found to be significantly associated with the occurrence of LR bacteria (import of animals, import country, age of the building, region, housing type, or organic production).

### 2.6. Follow-Up Analyses

A subsequent investigation in sows was prompted by the initial observation of an increased odds ratio for LR associated with general antimicrobial use (excluding florfenicol use). A follow-up analysis in sows showed a significant association with LR for lincomycin use (OR: 4.6, 95%CI: 1.05–20.31, *p*-value: 0.04) and ampicillin use (OR: 10.46, 95%CI: 1.57–114.57, *p*-value: 0.02). Among the eight herds with sows that reported lincomycin use, four were reported to carry LR bacteria. The whole-genome sequence (WGS) analysis [3] of these four latter isolates suggested that lincomycin use could cross-select the LR in one *Staphylococcus sciuri* isolate through the detection of the *cfr* gene encoding a.o. linezolid and lincomycin resistance. In the remaining three isolates, lincomycin use could co-select the LR *optrA* gene either through the co-location of an *erm(A)* gene on the same contig (n = 1; *Enterococcus faecalis* with *optrA*-ORG-5 genetic organization [3]) or the carriage of other lincomycin resistance genes elsewhere in the genome: *erm(B)* in one *Enterococcus faecium* isolate or *erm(B)*, *lnu(B)*, *lsa(E)* and *lsa(A)* in one *E. faecalis* isolate.

Among the four herds with sows that reported ampicillin use, three were reported to carry LR bacteria (two *E. faecium* and one *S. aureus* bacterial isolates). The investigation of WGS data revealed no ampicillin resistance gene in the two *E. faecium* isolates and, therefore, no putative co-selection through the use of ampicillin. The last LR *S. aureus* isolates carried a.o., the *mecA* and *blaZ* beta-lactam resistance genes, and the *cfr* gene. In this isolate, the *cfr* gene could be either cross-selected with the other antibiotics associated with this resistance gene or through co-selection with beta-lactam antibiotics.

## 3. Discussion

To the best of our knowledge, this is the first study that explores putative risk factors for the occurrence of linezolid-resistant (LR) bacteria in food-producing animals.

The findings indicate that the use of florfenicol may increase the likelihood of LR bacteria in veal calves (OR: 3.19), fattening pigs (OR: 5.29 in fecal samples and 9.09 in nasal swab samples), and sows (OR: 6.11). This likelihood is supported by the presence of genes *cfr*, *optrA,* and/or *poxtA*, which are known to confer cross-resistance to phenicols and linezolid [8,9,10] in the resistant herds. Moreover, because genetic characterization revealed that *optrA* is the predominant gene among the collected LR isolates [3], and because this gene, unlike *cfr* and *poxtA*, confers resistance solely to linezolid and phenicols, we can assume that antimicrobials other than phenicols are less involved in this co/cross-selection. Indeed, in isolates carrying only the *optrA* gene, other antimicrobials might only be involved in the co-selection of this gene via other resistance genes carried on the same genetic element as *optrA*, while phenicol use could directly cross-select the *optrA* gene. The possibility of the cross/co-selection of LR using other antimicrobials (such as phenicols) highlights the importance of minimizing antimicrobial use whenever possible.

Among the LRP herds kept in this study (N = 111), two herds of laying hens carried LR bacteria without LR genes but with LR-conferring mutations in 23S rRNA that, to our knowledge [3], do not confer resistance to phenicols. In these two laying hen herds, no use of phenicols was recorded. These mutations may have occurred either spontaneously or under selective pressure, but the context of such selective pressure, if any, could not be deciphered with our data.

General antimicrobial use excluding florfenicol use was also found to be positively associated with LR in sows (OR: 10.07). Other antimicrobials, such as lincomycin, could be involved in the cross/co-selection of LR, as indicated by the follow-up analyses. Accordingly, a recent study in Germany reported the use of lincomycin as another possibility for the co-selection of the LR MRSA strains in their study, as both strains harbored *lsa* and *lnu* genes [45].

Interestingly, low levels of LR bacteria were found in the studied laying hen and broiler flocks, even though no or very little recent use of phenicols was recorded in the farms of these flocks (0.0% of laying hens flocks and 0.5% of broiler flocks with florfenicol use). It is most likely that other factors may play a role in the occurrence, maintenance, and spread of LR bacteria (e.g., routines for the disinfection of the floor between production cycles, the number of parent flocks supplying the broiler flock with day-old chickens [23] or the use of other antimicrobials (the mechanism of co-selection by other antimicrobials due to the presence of antimicrobial resistance (AMR) genes, as previously suspected for livestock-associated (LA)-MRSA [46,47])). In addition, future studies should address the potential role of animal movements in the spread of LR genes/bacteria between poultry farms since poultry movements are now registered in the national database.

Purchasing animals from other farms was positively associated with the occurrence of LR bacteria in sows (OR: 7.28). Pig-fattening herds (OR: 3.31), breeding herds (OR: 44.14), and piglet-rearing herds (OR: 44.14) were more likely to carry LR isolates compared to closed herds. This result is likely explained because closed herds, in theory, receive no animal introductions, and therefore, LR bacteria from other farms cannot be introduced in the farm through the arrival of new animals. These findings suggest that limiting the number of source farms and lowering the frequency of purchases could prevent the risk of spread of resistance between farms, although further research is required to confirm this hypothesis. Animal movements are an important driver in the spread of infectious diseases in livestock [48,49] and could play a similar role in the transmission of resistant bacteria and AMR genes. These findings are in line with previous studies demonstrating the role of pig movements in the spread of livestock-associated MRSA CC398 within the pig production system in Denmark [50] and the Netherlands [51,52].

The presence of other animal species (bovine, sheep, goat, poultry) on the pig farm reduced the odds for LR compared to the absence of other animal species by approximately 59% (OR: 0.41). This result was unexpected, considering we hypothesized that possible contacts between the pigs and other animal species (potentially carrying LR bacteria) might increase the risk of LR because the use of antimicrobials in these other animal species could also have selected the LR bacteria. Alternatively, a dilution effect of (detected) resistance and a fitness profit by more susceptible bacteria could have been present because of the putative greater diversity in the overall microbiome present in such multi-species settings. A study from Italy reported that both the number of swine and the number of swine herds close to dairy farms were positively associated with the occurrence of MRSA in dairy herds [53]. In another study, no association was found between the presence of pigs or other farm animals and the occurrence of MRSA in dairy herds in Northern Italy [54]. Both our results and the results of these studies in bovine herds illustrated that different specific situations could occur.

Our study has some limitations. Firstly, the lack of a significant association between LR and certain factors and in certain animal species (for example, the purchase of animals in veal calves) could be due to the small number of events. The small number of events did not allow for multivariate analyses, and therefore, we could not control for confounding factors. Moreover, obtaining precise estimates of the odds ratio was challenging in some instances, particularly when there were few events in some of the comparison groups (e.g., breeding and piglet-rearing herds). The sample size was defined a priori for the monitoring of AMR in MRSA and enterococci from food-producing animals and the selective monitoring of LR as part of the One Health European Joint Programme (OH-EJP) LIN-RES project [3]. Yet, this sample size was (at that time) not foreseen to allow the detection of differences in exposure among the LRP and LRN groups, which was added as a purpose later on in the project based on the findings of resistance presence and indications for a potential link with florfenicol use [3]. Consequently, the findings regarding exposure among LRP and LRN groups from this study should be interpreted carefully, as our study may have reduced power to detect statistically significant associations.

Another limitation is related to the resistance status of the herds which was based on the LR selective screening of a limited number of samples from a single farm and may have led to an underestimation of the true number of resistant herds. By collecting a limited number of samples from a farm (at a given time), resistance may be missed, especially in herds with a low prevalence of resistance. On the other hand, given that some samples were taken at slaughterhouses and not directly at the farms, contamination with AMR bacteria during transport or at slaughterhouses could not be excluded. The influence of phenicol use and other factors on the acquisition and persistence of LR in food-producing animals should be further assessed in future prospective longitudinal studies with more animals sampled per farm.

Furthermore, future studies should also consider the possible transfer of LR genes or the bacteria carrying them from surrounding environments to livestock. A recent study in China found that levels of florfenicol used on swine farms and the application of swine manure to soil could promote the accumulation of florfenicol resistance genes in soil adjacent to these farms, suggesting that soil may act as a reservoir for florfenicol resistance [55]. Additionally, LR genes-harboring enterococci have been found in various environmental settings [56,57,58]. This underscores the need for a One Health approach to fully understand and address the transmission dynamics of LR genes.

The investigation of detailed antimicrobial use data could provide further insights into the effect of the administration of antimicrobials to livestock on the risk of LR in commensal bacteria. In fact, not only could the dose of antimicrobials pose a risk for the development of antimicrobial resistance, but also the administration route (oral versus injection) [59]. For instance, a systematic review has shown that oral administration of antimicrobials increases the risk of AMR in *Escherichia coli* in swine [35].

## 4. Materials and Methods

This cross-sectional study was part of a larger research project within the framework of the One Health–European Joint Project (OH-EJP) and aimed to assess the occurrence of LR enterococci and staphylococci in food-producing animals in 2019 in Belgium (“LIN-RES”) [3].

### 4.1. Study Design

Aiming to identify the potential risk factors involved in the occurrence of LR bacteria in food-producing animals, first, a literature search and expert consultation were performed. Next, data on phenotypic LR in sampled herds were gathered. Afterwards, specific data on the identified putative risk factors were collected for the sampled population and their association with the presence of LR enterococci, staphylococci, and one *P. pentosaceus*, as established in the “LIN-RES”-project [3], was evaluated through univariate logistic regression analysis.

### 4.2. Potential Risk Factors

A list of putative risk factors was compiled by considering data from national database records with information on the animals, the holding, and the antibiotic use of the herds included in our study. A panel of experts, consisting of four members representing the veterinary fields of AMR (CB, MC, MEF, MT), veterinary epidemiology (MC, MEF), as well as veterinary bacteriology (CB, MT), was consulted during virtual face-to-face meetings and email correspondence to ask their opinion on these risk factors.

Afterward, we conducted a literature search to support the opinion of the experts and to verify if any putative risk factors had been overlooked. The database PubMed was consulted in March 2022, considering a 10-year inclusion period and species defined as “Other animals”, using the following keywords and their combinations in the title and abstract: calves, pigs, broiler, laying hen/layer hen, risk factor, antimicrobial use/antibiotic use, and resistance (Table S1 in Supplementary Materials). Additional pertinent publications referenced by the identified papers were manually searched. The following factors were excluded due to a lack of available data: the predominant animal breed of the herd, hygiene and biosafety practices, the number of barns per flock, status of the previous flock in the broiler house, bio-label (organic versus conventional production), the housing of calves (type of stable), age of the calves and feeding of calves with milk or colostrum treated with antimicrobials.

### 4.3. Study Population

The study population for the feces examinations consisted of 247 herds with fattening pigs, 190 broiler flocks, 179 laying hen flocks, and 113 herds with veal calves, and for the analysis in nasal swabs, 77 herds with sows and 69 herds with fattening pigs.

#### 4.3.1. Sample Collection

A total of 1325 fecal samples (broilers n  =  295, turkeys n  =  86, laying hens n  =  206, breeding hens n  =  163, veal calves n  =  292 and pigs n  =  283) and 148 nasal swab samples (sows n  =  78 and fattening pigs n  =  70) were collected in 2019 from healthy animals for the Belgian official monitoring of AMR for MRSA and enterococci from food-producing animals [60]. Samples were taken by official agents throughout the year, using a randomized sampling design, in adherence with the provisions of the EU decision 2013/652/EU [61] and the technical specifications issued by EFSA [62] in the framework of the national control programme of the Federal Agency for the Safety of the Food Chain (FASFC) for the official monitoring of AMR. The FASFC ensured the randomization of the sampling scheme and its implementation. The random sampling plan was stratified per slaughterhouse by allocating the number of samples collected per slaughterhouse proportionally to the annual throughput of the slaughterhouse, as specified by EFSA [62]. Similarly, a random sampling plan on the farm was stratified according to the number of farms per animal category per local control unit. The fecal samples for the monitoring of enterococci in veal calves, pigs, and broilers were collected at slaughterhouses. The epidemiological unit for veal caves and pigs was the slaughter batch, defined as a batch of animals sent to the slaughterhouse at the same moment. Only one animal was sampled per epidemiological unit. Regarding broilers, ten animals per epidemiological unit, defined as the flock, were sampled and pooled. The nasal swab samples for the monitoring of MRSA in pigs were collected on the farm (ten animals per holding were sampled and pooled). Thus, nasal swab samples and fecal samples from fattening pigs were not collected on the same animals nor from the same sampling place (farm vs. slaughterhouse). The monitoring of enterococci in laying hen flocks was a form of supplementary monitoring not included in the EU decision, with samples being taken on the farm (multiple animals without count per epidemiological unit, defined as the flock). Fecal samples from turkeys and breeding hens collected for the AMR official monitoring were excluded from the present study due to the absence of data on antimicrobial use in the Sanitel-Med database (Federal Agency for Medicines and Health Products, FAMHP), as the registration of antimicrobial use in these animal species was not yet required by law.

#### 4.3.2. Isolation of LR Enterococci and Staphylococci

After official monitoring analysis [60], all bacteria grown on Petri dishes from the official AMR monitoring of MRSA and enterococci were collected and spread on Columbia Sheep Blood (CSB) supplemented with linezolid (4 mg/L) for phenotypic LR selection, as described and reported by Timmermans et al. [3]. The isolated LR bacteria were enterococci and staphylococci [3]. By chance, one *P. pentosaceus* was recovered through this isolation method and kept for the LR status assignment.

#### 4.3.3. LR Status of a Herd

A herd was classified as LRP if phenotypic LR was observed in at least one sample taken from that herd. A herd was classified as LRN if all samples taken from different epidemiological units of the herd, as described by EFSA, were negative for phenotypic LR. Samples that could not be linked to a herd identification number (Sanitel ID unknown) in the Belgian national livestock database Sanitel (FASFC) were excluded.

The number of herds finally kept in the study for the feces examination consisted of 247 herds with fattening pigs, 190 broiler flocks, 179 laying hen flocks, and 113 herds with veal calves, and for the analysis in nasal swabs, 77 herds with sows and 69 herds with fattening pigs.

#### 4.3.4. Whole-Genome Sequencing

The WGS analysis was conducted previously, as described by Timmermans et al. [3] and deposited in SRA under BioProject PRJNA670413. Briefly, genomic DNA was extracted using the DNeasy Blood and Tissue kit according to the manufacturer’s instructions (Qiagen, Hilden, Germany). Isolate sequencing libraries were created using Nextera XT DNA library preparation (Illumina, San Diego, CA, USA) according to the manufacturer’s instructions and were subsequently sequenced using MiSeq V3 chemistry (Illumina) for the production of 2 × 250 bp paired-end reads. Reads were trimmed, and de novo assembled, as described by Bogaerts et al. [63]. The detection of LR genes was performed as described for gene detection by Bogaerts et al. [63] using the sequences from the LRE-Finder database [64]. Hits with ≤90% sequence identity or ≤90% target coverage were removed. The same methodology was used to detect other AMR genes using the ResFinder database [65]. These WGS data were used to investigate further the potential genetic background of cross/co-selection with other antibiotic use that could support the associations found in sows (i.e., association with LR for lincomycin use and ampicillin use). This investigation consisted of the detection of AMR genes and their position in contigs, as described above, and in LR bacteria isolated from herds with reported lincomycin or ampicillin use.

### 4.4. Collection of Risk Factor Data

#### 4.4.1. Antimicrobial Use

In order to assess the use of antimicrobials, farm-level antimicrobial use data in veal calves, broilers, laying hens, and pigs in Belgium between 1 March 2017, and 31 December 2019, available in the Sanitel-Med database (FAMHP), were provided by AMCRA. Based on the date of registration, the use of antimicrobials by the farm was expressed over the estimated minimum average period of stay of animals on the farm before sampling. The average stay of an animal on the farm until sampling was defined for each combination of sample types and animal categories based on expert opinion (experts from the team of porcine health at the Faculty of Veterinary Medicine (Ghent University) and FASFC) and/or available animal movement data: four months for veal calves, three months for fattening pigs sampled at slaughter (fecal samples), one and a half month for fattening pigs sampled on farms (nasal swab samples), one year for sows and one month for broilers and laying hens. When calculating the average duration of stay per farm or flock using animal movement data, the minimum value across all farms was taken to ensure that only antimicrobial use during the period when the animals sampled were present in each farm was considered.

#### 4.4.2. Animal Movements

Animal movement data covering the period between 1 January 2017, and 31 December 2019, were acquired from the Belgian national livestock identification register (Sanitel, FASFC). These records contain, among others, the identification (ID) numbers of both the source and destination herds, the date of movement, and for bovine animals, the ID of the animal, and, when applicable, the country of origin. Bovine movements encompassed both between-farm transfers and the import movements of individual animals, while movements through markets were not considered. Pig movements exclusively consisted of transfers of batches of animals between farms, as pig imports were not systematically registered in Sanitel and, therefore, excluded from the analysis. The mandatory registration of poultry movements was effectively applied from 1 February 2019 [66] (after the start of the sampling), and consequently, these movements were not considered in the analysis. Furthermore, only the movements that occurred in the above-estimated minimum average period that these animals remained on a farm before sampling were taken into account.

#### 4.4.3. Farm and Herd Data

The Sanitel database was queried to extract information related to the holding and the herd. Data regarding the holding consisted of province name and housed animal species, while herd-level data included herd type, production type, inventory, capacity, counts, official veterinarian, registration date, and type of housing. Inventory data (available in the Sanitel data warehouse within Sciensano) were used to calculate bovine herd size, count data were used to estimate pig herd size, and capacity data were used to estimate poultry herd size. The age of the establishment at the beginning of the study period (2019) was computed from the date of registration. Independent categorical variables with few observations per category were aggregated when the creation of new, more relevant categories was possible (organic production, number of farm animal species). When a variable had many categories and grouping categories into larger meaningful categories was not possible (e.g., veterinarian), the variable was not used in the analysis.

### 4.5. Statistical Analysis

Data were analyzed using the statistical software R version 4.0.4 [67]. Descriptive statistics were used to describe the features of the LRP and LRN herds. Categorical variables were described in absolute frequencies and percentages, and continuous variables were described as the median, first and third quartiles, mean, and standard deviation. The associations of potential predictor variables (i.e., risk factors) (Table 1) with the occurrence of LR (odds ratio, OR) were examined by univariate logistic regression models. Herds with missing data on a certain variable were excluded from the univariate analysis of that variable. Firth’s penalized likelihood approach was applied to address small sample bias [68,69,70]. A major advantage of Firth’s estimation is that, unlike maximum likelihood estimation, it provides useful (finite) estimates in the case of data separation. Confidence intervals were computed by the penalized profile likelihood. Independent variables were screened for univariate associations using the likelihood ratio test statistic. Statistical significance was evaluated at a *p*-value of ≤0.05.

## 5. Conclusions

In conclusion, the present study revealed that, in veal calves (feces), fattening pigs (feces and nasal swabs), and sows (nasal swabs), the use of florfenicol increases the odds of finding LR bacteria collected from feces and nasal swabs. Furthermore, in sows, general antimicrobial use and the purchase of animals were found to increase the odds of finding LR bacteria when collected from nasal swabs. Follow-up analyses in sows indicated that other antimicrobials, such as lincomycin, could also be involved in the cross/co-selection of LR. Also, in fattening pigs, herd types associated with animal introductions were more likely to carry LR bacteria compared to closed herds, and herds with the presence of other animal species on the farm were less likely to carry LR bacteria compared to herds without other species. Preventive measures should consist of improving the appropriate use of antimicrobials and biosecurity on farms. This could be achieved by limiting the use of phenicols and other antimicrobials (a.o. lincosamides, pleuromutilins, and tetracyclines) to avoid the cross/co-selection of LR genes. External biosecurity could be improved by screening source farms or limiting the number of source farms for purchase to avoid the potential spread of resistance genes between farms.

In the future, longitudinal studies with several periods of sampling and with fit-for-purpose sampling, enabling the follow-up of individual animals for both antimicrobial use and resistance, should be performed in order to better understand the risk factors for the occurrence of LR in food-producing animals. Additionally, the environmental transmission of LR to animals should be considered in future studies. Furthermore, the role of food-producing animals as a source of LR for humans in current European production systems should be elucidated. Adopting a One Health approach is essential to elucidate the interconnected roles of food-producing animals and environmental reservoirs in the transmission of LR to humans.

## Figures and Tables

**Table 1:** Potential risk factors for LR investigated in this study.

Risk Factor	Type of Variable	Rationale	Reference	Animal Species Examined in This Study
Purchase of animals during a defined period prior to sampling	Categorical (binary(Yes/No))	LR can potentially be introduced in a herd via the purchase of carrier animals within Belgium	[19,20,21,22,23,24,25], expert opinion	Veal calves, pigs
Import of animals from outside Belgium	Categorical (binary(Yes/No))	LR can potentially be introduced in a herd via the purchase of carrier animals from outside Belgium	expert opinion	Veal calves
Import country	Categorical (No import/Country A/Country B/Country C) ^a^	LR can potentially be introduced in a herd via the purchase of carrier animals from outside Belgium; the risk of introducing LR can potentially vary by import country	[26], expert opinion	Veal calves
Production type	Categorical (Closed/Fattening/Breeding/Mixed/Rearing/Piglets rearing) ^b^	Susceptibility and herd management may differ between categories	[27], expert opinion	Pigs
Herd size	Continuous	Larger herd size could be associated with more contact between animals and increased risk for antimicrobial resistance transmission due to direct and indirect contact	[20,22], expert opinion	Veal calves, pigs, poultry
Age of the building (best available proxy for biosecurity level)	Continuous	Higher biosecurity levels and less morbidity are expected in more recent farms	expert opinion	Veal calves, pigs, poultry
Housing type	Categorical (Organic, Outdoor, Enriched cage, Free-range)	Organic means less use of antimicrobials, access outside, and more space allowance per animal and thus less contact between animals	[22,28], expert opinion	Poultry
Organic	Categorical (Yes/No)	Organic means less use of antimicrobials, access outside, and more space allowance per animal and thus less contact between animals	[22,28], expert opinion	Poultry
Florfenicol use	Categorical (binary(Yes/No))	Selection of resistance genes conferring cross-resistance to linezolid	[3], expert opinion	Veal calves, pigs, poultry
Antimicrobial use (all antimicrobials except florfenicol)	Categorical (binary(Yes/No))	Comorbidity (other diseases and antibiotic treatment) and potential co- or cross-selection of LR genes	[20,29,30,31,32,33,34,35,36,37,38,39,40,41,42,43,44], expert opinion	Veal calves, pigs, poultry
Region	Categorical (Region A/Region B) ^a^	Geographic location of the farm	[27], expert opinion	Veal calves, pigs, poultry
Number of farm animal species	Categorical (1 (absence of other animal species) or >1 (presence of other animal species) in pigs and poultry and 1 (absence of other animal species), 2 (one other animal species) or 3 (two other animal species) in veal calves)	The presence of other livestock animal species on the farm	[20,28], expert opinion	Veal calves, pigs, poultry

^a^ Country A/B/C and Region A/B: these letter codes are used to guarantee anonymity. ^b^ In Belgium, seven types of pig herds are defined in accordance with the Royal Decree 2014/11434: Closed herds are herds with a capacity for reproducers, piglets, and fattening pigs (often called farrow-to-finisher herds). Fattening herds produce finishers that are sent to slaughterhouses. Breeding herds are reproduction herds that produce piglets that are transferred to piglet-rearing herds. Companion herds are herds with less than 3 animals for non-commercial use. Piglet-rearing herds only contain piglets. Mixed herds are herds that do not enter into only one of the previous categories.

**Table 2:** Number of herds (N, %) within linezolid-resistant-positive (LRP) and linezolid-resistant-negative (LRN) groups per livestock species for each of the investigated categorical and continuous variables (fecal samples).

		Number of Herds (N, %) per Species											
		Veal Calves			Fattening Pigs			Laying Hens			Broilers		
Variable	Category	LRP	LRN	Total	LRP	LRN	Total	LRP	LRN	Total	LRP	LRN	Total
		(N = 31)	(N = 82)	(N = 113)	(N = 31)	(N = 216)	(N = 247)	(N = 3)	(N = 176)	(N = 179)	(N = 13)	(N = 177)	(N = 190)
**Demographics**													
Herd size	Mean (SD)	810.40 (518.05)	687.12 (343.18)	720.94 (400.14)	1849.44 (1844.22)	1493.46 (1437.91)	1538.14 (1495.25)	15,400.00 (17,328.59)	28,327.05 (30,978.77)	28,110.39 (30,816.44)	20,130.77 (7020.61)	30,561.29 (23,286.94)	29,847.63 (22,695.43)
	Median (1st Qu, 3rd Qu)	645.00 (538.50, 818.75)	601.50 (497.62, 819.25)	622.50 (498.00, 819.50)	1463.50 (735.75, 1947.50)	1133.25 (593.12, 1838.38)	1178.00 (607.75, 1856.50)	8000.00 (5500.00, 21,600.00)	19,990.00 (6000.00, 36,241.00)	19,990.00 (6000.00, 36,000.00)	21,000.00 (18,000.00, 23,000.00)	25,000.00 (19,900.00, 39,000.00)	24,000.00 (19,562.50, 36,000.00)
Number of farm animal species	1	26 (83.9%)	66 (80.5%)	92 (81.4%)	25 (80.6%)	133 (61.6%)	158 (64.0%)	2 (66.7%)	108 (61.4%)	110 (61.5%)	4 (30.8%)	91 (51.4%)	95 (50.0%)
	>1	N.A.	N.A.	N.A.	6 (19.4%)	83 (38.4%)	89 (36.0%)	1 (33.3%)	68 (38.6%)	69 (38.5%)	9 (69.2%)	86 (48.6%)	95 (50.0%)
	2	3 (9.7%)	12 (14.6%)	15 (13.3%)	N.A.	N.A.	N.A.	N.A.	N.A.	N.A.	N.A.	N.A.	N.A.
	3	2 (6.5%)	4 (4.9%)	6 (5.3%)	N.A.	N.A.	N.A.	N.A.	N.A.	N.A.	N.A.	N.A.	N.A.
Region	Region A	31 (100%)	77 (93.9%)	108 (95.6%)	29 (93.5%)	184 (85.2%)	213 (86.2%)	2 (66.7%)	120 (68.2%)	122 (68.2%)	8 (61.5%)	145 (81.9%)	153 (80.5%)
	Region B	0 (0%)	5 (6.1%)	5 (4.4%)	2 (6.5%)	32 (14.8%)	34 (13.8%)	1 (33.3%)	56 (31.8%)	57 (31.8%)	5 (38.5%)	32 (18.1%)	37 (19.5%)
**Husbandry types**													
Production type	Closed	N.A.	N.A.	N.A.	3 (9.7%)	51 (23.6%)	54 (21.9%)	N.A.	N.A.	N.A.	N.A.	N.A.	N.A.
	Mixed	N.A.	N.A.	N.A.	5 (16.1%)	70 (32.4%)	75 (30.4%)	N.A.	N.A.	N.A.	N.A.	N.A.	N.A.
	Fattening	N.A.	N.A.	N.A.	21 (67.7%)	95 (44.0%)	116 (47.0%)	N.A.	N.A.	N.A.	N.A.	N.A.	N.A.
	Breeding	N.A.	N.A.	N.A.	1 (3.2%)	0 (0%)	1 (0.4%)	N.A.	N.A.	N.A.	N.A.	N.A.	N.A.
	Piglets rearing	N.A.	N.A.	N.A.	1 (3.2%)	0 (0%)	1 (0.4%)	N.A.	N.A.	N.A.	N.A.	N.A.	N.A.
	Rearing	N.A.	N.A.	N.A.	0 (0%)	0 (0%)	0 (0%)	N.A.	N.A.	N.A.	N.A.	N.A.	N.A.
Housing type	Organic	N.A.	N.A.	N.A.	N.A.	N.A.	N.A.	0 (0%)	58 (33.0%)	58 (32.4%)	0 (0%)	7 (4.0%)	7 (3.7%)
	Enriched cage	N.A.	N.A.	N.A.	N.A.	N.A.	N.A.	1 (33.3%)	31 (17.6%)	32 (17.9%)	0 (0%)	0 (0%)	0 (0%)
	Free-range	N.A.	N.A.	N.A.	N.A.	N.A.	N.A.	1 (33.3%)	55 (31.3%)	56 (31.3%)	0 (0%)	0 (0%)	0 (0%)
	Not specified	N.A.	N.A.	N.A.	N.A.	N.A.	N.A.	0 (0%)	3 (1.7%)	3 (1.7%)	7 (53.8%)	139 (78.5%)	146 (76.8%)
	Outdoor	N.A.	N.A.	N.A.	N.A.	N.A.	N.A.	1 (33.3%)	29 (16.5%)	30 (16.8%)	0 (0%)	0 (0%)	0 (0%)
	Missing	N.A.	N.A.	N.A.	N.A.	N.A.	N.A.	0 (0%)	0 (0%)	0 (0%)	6 (46.2%)	31 (17.5%)	37 (19.5%)
Organic	Yes	N.A.	N.A.	N.A.	N.A.	N.A.	N.A.	0 (0%)	58 (33.0%)	58 (32.4%)	0 (0%)	7 (4.0%)	7 (3.7%)
	No	N.A.	N.A.	N.A.	N.A.	N.A.	N.A.	3 (100%)	118 (67.0%)	121 (67.6%)	7 (53.8%)	139 (78.5%)	146 (76.8%)
	Missing	N.A.	N.A.	N.A.	N.A.	N.A.	N.A.	0 (0%)	0 (0%)	0 (0%)	6 (46.2%)	31 (17.5%)	37 (19.5%)
**Biosecurity**													
Age of the building in years	Mean (SD)	20.97 (1.66)	19.98 (5.00)	20.2 (4.36)	23.71 (4.16)	23.16 (5.07)	23.23 (4.96)	8.00 (10.39)	13.61 (8.44)	13.51 (8.47)	15.62 (8.35)	14.71 (8.52)	14.77 (8.49)
	Median (1st Qu, 3rd Qu)	21.00 (15.00, 22.00)	22.00 (2.00, 27.00)	22.00 (2.00, 27.00)	25.00 (25.00, 25.00)	25.00 (25.00, 25.00)	25.00 (25.00, 25.00)	2.00 (2.00, 11.00)	19.50 (3.00, 21.00)	19.00 (3.00, 21.00)	21.00 (7.00, 21.00)	21.00 (4.00, 21.00)	21.00 (4.00, 21.00)
Purchase of animals	No	19 (61.3%)	64 (78.0%)	83 (73.5%)	17 (54.8%)	115 (53.2%)	132 (53.4%)	N.A.	N.A.	N.A.	N.A.	N.A.	N.A.
	Yes	12 (38.7%)	18 (22.0%)	30 (26.5%)	14 (45.2%)	101 (46.8%)	115 (46.6%)	N.A.	N.A.	N.A.	N.A.	N.A.	N.A.
Import of animals	No	30 (96.8%)	78 (95.1%)	108 (95.6%)	N.A.	N.A.	N.A.	N.A.	N.A.	N.A.	N.A.	N.A.	N.A.
	Yes	1 (3.2%)	4 (4.9%)	5 (4.4%)	N.A.	N.A.	N.A.	N.A.	N.A.	N.A.	N.A.	N.A.	N.A.
Import country	No import	30 (96.8%)	78 (95.1%)	108 (95.6%)	N.A.	N.A.	N.A.	N.A.	N.A.	N.A.	N.A.	N.A.	N.A.
	Country A	0 (0%)	1 (1.2%)	1 (0.9%)	N.A.	N.A.	N.A.	N.A.	N.A.	N.A.	N.A.	N.A.	N.A.
	Country B	1 (3.2%)	2 (2.4%)	3 (2.7%)	N.A.	N.A.	N.A.	N.A.	N.A.	N.A.	N.A.	N.A.	N.A.
	Country C	0 (0%)	1 (1.2%)	1 (0.9%)	N.A.	N.A.	N.A.	N.A.	N.A.	N.A.	N.A.	N.A.	N.A.
**Antibiotic use**													
Florfenicol use	No	9 (29.0%)	46 (56.1%)	55 (48.7%)	15 (48.4%)	182 (84.3%)	197 (79.8%)	3 (100%)	154 (87.5%)	157 (87.7%)	13 (100%)	176 (99.4%)	189 (99.5%)
	Yes	22 (71.0%)	34 (41.5%)	56 (49.6%)	15 (48.4%)	34 (15.7%)	49 (19.8%)	0 (0%)	0 (0%)	0 (0%)	0 (0%)	1 (0.6%)	1 (0.5%)
	Missing	0 (0%)	2 (2.4%)	2 (1.8%)	1 (3.2%)	0 (0%)	1 (0.4%)	0 (0%)	22 (12.5%)	22 (12.3%)	0 (0%)	0 (0%)	0 (0%)
Other antibiotic use	No	3 (9.7%)	12 (14.6%)	15 (13.3%)	7 (22.6%)	74 (34.3%)	81 (32.8%)	3 (100%)	147 (83.5%)	150 (83.8%)	4 (30.8%)	58 (32.8%)	62 (32.6%)
	Yes	28 (90.3%)	68 (82.9%)	96 (85.0%)	23 (74.2%)	142 (65.7%)	165 (66.8%)	0 (0%)	7 (4.0%)	7 (3.9%)	9 (69.2%)	119 (67.2%)	128 (67.4%)
	Missing	0 (0%)	2 (2.4%)	2 (1.8%)	1 (3.2%)	0 (0%)	1 (0.4%)	0 (0%)	22 (12.5%)	22 (12.3%)	0 (0%)	0 (0%)	0 (0%)

N.A.: not applicable. SD: standard deviation. LRP: linezolid resistance positive. LRN: linezolid resistance negative. Country A/B/C and Region A/B: these letter codes are used to guarantee anonymity. Other antibiotic use means “antibiotic use (minus florfenicol use)”.

**Table 3:** Number of herds (N, %) within linezolid-resistant-positive (LRP) and linezolid-resistant-negative (LRN) groups per livestock species for each of the investigated categorical and continuous variables (nasal swab samples).

		Number of Herds (N, %) per Species					
		Fattening Pigs			Sows		
Variable	Category	LRP	LRN	Total	LRP	LRN	Total
		(N = 17)	(N = 52)	(N = 69)	(N = 16)	(N = 61)	(N = 77)
**Demographics**							
Herd size	Mean (SD)	950.97 (426.43)	1052.86 (1001.29)	1027.75 (892.57)	1771.66 (1230.68)	1485.40 (1243.10)	1544.88 (1237.97)
	Median (1st Qu, 3rd Qu)	950.50 (530.00, 1333.00)	704.50 (398.12, 1372.50)	742.00 (449.50, 1366.00)	1512.50 (903.25, 2089.75)	1265.00 (665.00, 1941.50)	1283.00 (683.50, 1990.00)
Number of farm animal species	1	8 (47.1%)	31 (59.6%)	39 (56.5%)	10 (62.5%)	36 (59.0%)	46 (59.7%)
	>1	9 (52.9%)	21 (40.4%)	30 (43.5%)	6 (37.5%)	25 (41.0%)	31 (40.3%)
Region	Region A	14 (82.4%)	47 (90.4%)	61 (88.4%)	16 (100%)	59 (96.7%)	75 (97.4%)
	Region B	3 (17.6%)	5 (9.6%)	8 (11.6%)	0 (0%)	2 (3.3%)	2 (2.6%)
**Husbandry types**							
Production type	Closed	0 (0%)	4 (7.7%)	4 (5.8%)	4 (25.0%)	15 (24.6%)	19 (24.7%)
	Mixed	2 (11.8%)	10 (19.2%)	12 (17.4%)	11 (68.8%)	39 (63.9%)	50 (64.9%)
	Fattening	15 (88.2%)	37 (71.2%)	52 (75.4%)	0 (0%)	3 (4.9%)	3 (3.9%)
	Breeding	0 (0%)	0 (0%)	0 (0%)	1 (6.3%)	4 (6.6%)	5 (6.5%)
	Piglets rearing	0 (0%)	0 (0%)	0 (0%)	0 (0%)	0 (0%)	0 (0%)
	Rearing	0 (0%)	1 (1.9%)	1 (1.4%)	0 (0%)	0 (0%)	0 (0%)
**Biosecurity**							
Age of the building in years	Mean (SD)	23.73 (4.51)	23.53 (4.16)	23.68 (4.40)	25.00 (0.00)	24.54 (3.11)	24.64 (2.77)
	Median (1st Qu, 3rd Qu)	25.00 (25.00, 25.00)	25.00 (25.00, 25.00)	25.00 (25.00, 25.00)	25.00 (25.00, 25.00)	25.00 (25.00, 25.00)	25.00 (25.00, 25.0)
Purchase of animals	No	10 (58.8%)	36 (69.2%)	46 (66.7%)	2 (12.5%)	34 (55.7%)	36 (46.8%)
	Yes	7 (41.2%)	16 (30.8%)	23 (33.3%)	14 (87.5%)	27 (44.3%)	41 (53.2%)
**Antibiotic use**							
Florfenicol use	No	11 (64.7%)	47 (90.4%)	58 (84.1%)	11 (68.8%)	57 (93.4%)	68 (88.3%)
	Yes	5 (29.4%)	2 (3.8%)	7 (10.1%)	5 (31.3%)	4 (6.6%)	9 (11.7%)
	Missing	1 (5.9%)	3 (5.8%)	4 (5.8%)	0 (0%)	0 (0%)	0 (0%)
Other antibiotic use	No	5 (29.4%)	27 (51.9%)	32 (46.4%)	0 (0%)	14 (23.0%)	14 (18.2%)
	Yes	11 (64.7%)	22 (42.3%)	33 (47.8%)	16 (100%)	47 (77.0%)	63 (81.8%)
	Missing	1 (5.9%)	3 (5.8%)	4 (5.8%)	0 (0%)	0 (0%)	0 (0%)

Variables for which none of the categories applied were removed from the table (i.e., import of animals, import country, housing type, and organic). SD: standard deviation. LRP: linezolid resistance positive. LRN: linezolid resistance negative. Region A/B: these letter codes are used to guarantee anonymity. Other antibiotic use means “antibiotic use (minus florfenicol use)”.

**Table 4:** Results of the univariable logistic regression model assessing the association between putative risk factors and the occurrence of linezolid-resistant (LR) bacteria in feces of veal calves, fattening pigs, laying hens, and broilers.

		Livestock Species															
		Veal Calves				Fattening Pigs				Laying Hens				Broilers			
Variable	Category	n	OR	Lower–Upper 95% CI	*p*-Value	n	OR	Lower–Upper 95% CI	*p*-Value	n	OR	Lower–Upper 95% CI	*p*-Value	n	OR	Lower–Upper 95% CI	*p*-Value
**Demographics**																	
Herd size		113	1.00	[1.00–1.00]	0.15	247	1.00	[1.00–1.00]	0.19	179	1.00	[1.00–1.00]	0.92	190	1.00	[1.00–1.00]	**0.03**
Number of farm animal species	1		-	-	-		-	-	-		-	-	-		-	-	-
	>1		N.A.	N.A.	N.A.	247	0.41	[0.15–0.95]	**0.04**	179	0.95	[0.09–7.30]	0.96	190	2.23	[0.73–7.88]	0.16
	2	113	0.70	[0.17–2.30]	0.58		N.A.	N.A.	N.A.		N.A.	N.A.	N.A.		N.A.	N.A.	N.A.
	3	113	1.39	[0.23–6.71]	0.69		N.A.	N.A.	N.A.		N.A.	N.A.	N.A.		N.A.	N.A.	N.A.
Region	Region A		-	-	-		-	-	-		-	-	-		-	-	-
	Region B	113	0.22	[0.00–2.07]	0.22	247	0.48	[0.09–1.56]	0.25	179	1.28	[0.12–9.84]	0.82	190	2.90	[0.88–8.92]	0.08
**Husbandry types**																	
Production type	Closed		N.A.	N.A.	N.A.		-	-	-		N.A.	N.A.	N.A.		N.A.	N.A.	N.A.
	Mixed		N.A.	N.A.	N.A.	247	1.15	[0.29–5.14]	0.84		N.A.	N.A.	N.A.		N.A.	N.A.	N.A.
	Fattening		N.A.	N.A.	N.A.	247	3.31	[1.14–12.90]	**0.03**		N.A.	N.A.	N.A.		N.A.	N.A.	N.A.
	Breeding		N.A.	N.A.	N.A.	247	44.14	[1.99–6971.80]	**0.02**		N.A.	N.A.	N.A.		N.A.	N.A.	N.A.
	Piglets rearing		N.A.	N.A.	N.A.	247	44.14	[1.99–6971.80]	**0.02**		N.A.	N.A.	N.A.		N.A.	N.A.	N.A.
Housing type	Organic		N.A.	N.A.	N.A.		N.A.	N.A.	N.A.		-	-	-		-	-	-
	Enriched cage		N.A.	N.A.	N.A.		N.A.	N.A.	N.A.	179	5.57	[0.29–822.90]	0.25		N.A.	N.A.	N.A.
	Free-range		N.A.	N.A.	N.A.		N.A.	N.A.	N.A.	179	3.16	[0.17–465.45]	0.45		N.A.	N.A.	N.A.
	Not specified		N.A.	N.A.	N.A.		N.A.	N.A.	N.A.	179	16.71	[0.08–3332.97]	0.22	153	0.81	[0.08–108.30]	0.89
	Outdoor		N.A.	N.A.	N.A.		N.A.	N.A.	N.A.	179	5.95	[0.31–879.17]	0.24		N.A.	N.A.	N.A.
Organic	Yes		N.A.	N.A.	N.A.		N.A.	N.A.	N.A.		-	-	-		-	-	-
	No		N.A.	N.A.	N.A.		N.A.	N.A.	N.A.	179	3.46	[0.33–467.57]	0.35	153	0.81	[0.08–108.30]	0.89
**Biosecurity**																	
Age of the building		113	1.05	[0.95–1.21]	0.34	247	1.02	[0.95–1.12]	0.71	179	0.94	[0.81–1.05]	0.27	190	1.01	[0.95–1.09]	0.79
Purchase of animals	No		-	-	-		-	-	-		N.A.	N.A.	N.A.		N.A.	N.A.	N.A.
	Yes	113	2.23	[0.92–5.39]	0.08	247	0.94	[0.44–1.99]	0.88		N.A.	N.A.	N.A.		N.A.	N.A.	N.A.
Import of animals		113	0.86	[0.08–4.89]	0.87		N.A.	N.A.	N.A.		N.A.	N.A.	N.A.		N.A.	N.A.	N.A.
Import country	No import		-	-	-		N.A.	N.A.	N.A.		N.A.	N.A.	N.A.		N.A.	N.A.	N.A.
	Country A	113	0.86	[0.01–16.54]	0.92		N.A.	N.A.	N.A.		N.A.	N.A.	N.A.		N.A.	N.A.	N.A.
	Country B	113	1.54	[0.14–12.09]	0.69		N.A.	N.A.	N.A.		N.A.	N.A.	N.A.		N.A.	N.A.	N.A.
	Country C	113	0.86	[0.01–16.54]	0.92		N.A.	N.A.	N.A.		N.A.	N.A.	N.A.		N.A.	N.A.	N.A.
**Antibiotic use**																	
Florfenicol use	No		-	-	-		-	-	-		N.A.	N.A.	N.A.		-	-	-
	Yes	111	3.19	[1.36–7.97]	**0.01**	246	5.29	[2.39–11.78]	**<0.01**		N.A.	N.A.	N.A.	190	4.36	[0.03–85.88]	0.44
Other antibiotic use	No		-	-	-		-	-	-		-	-	-		-	-	-
	Yes	111	1.49	[0.46–6.14]	0.53	246	1.64	[0.72–4.16]	0.25	157	2.81	[0.02–33.25]	0.55	190	1.03	[0.34–3.66]	0.96

N.A.: not applicable. OR: odds ratio. CI: confidence interval. -: reference category. The rearing category for the variable “production type” was N.A. for all livestock species and is therefore not shown in this table. Country A/B/C and Region A/B: these letter codes are used to guarantee anonymity. Other antibiotic use means “antibiotic use (minus florfenicol use)”.

**Table 5:** Results of the univariable logistic regression model assessing the association between putative risk factors and the occurrence of LR bacteria in the nasal swabs of fattening pigs and sows.

		Livestock Species							
		Fattening Pigs				Sows			
Variable	Category	n	OR	Lower–Upper 95% CI	*p*-Value	n	OR	Lower–Upper 95% CI	*p*-Value
**Demographics**									
Herd size		69	1.00	[1.00–1.00]	0.81	77	1.00	[1.00–1.00]	0.37
Number of farm animal species	1		-	-	-		-	-	-
	>1	69	1.64	[0.56–4.90]	0.37	77	0.89	[0.28–2.63]	0.83
Region	Region A		-	-	-		-	-	-
	Region B	69	2.08	[0.44–8.89]	0.34	77	0.72	[0.01–9.45]	0.83
**Husbandry types**									
Production type	Closed		-	-	-		-	-	-
	Mixed	69	2.14	[0.13–319.24]	0.62	77	1.00	[0.30–3.78]	1.00
	Fattening	69	3.72	[0.36–504.15]	0.31	77	0.49	[0.00–6.70]	0.64
	Breeding		N.A.	N.A.	N.A.	77	1.15	[0.10–8.77]	0.90
	Rearing	69	3.00	[0.01–702.69]	0.62		N.A.	N.A.	N.A.
**Biosecurity**									
Age of the building		69	0.98	[0.88–1.11]	0.72	77	1.00	[0.88–1.45]	0.96
Purchase of animals	No		-	-	-		-	-	-
	Yes	69	1.58	[0.51–4.76]	0.42	77	7.28	[2.00–39.26]	**<0.01**
**Antibiotic use**									
Florfenicol use	No		-	-	-		-	-	-
	Yes	65	9.09	[1.92–56.15]	**0.01**	77	6.11	[1.51–26.25]	**0.01**
Other antibiotic use	No		-	-	-		-	-	-
	Yes	65	2.56	[0.83–8.70]	0.10	77	10.07	[1.22–1313.51]	**0.03**

N.A.: not applicable. Variables for which none of the categories applied were removed from the table (i.e., import of animals, import country, housing type, and organic). OR: odds ratio. CI: confidence interval. -: reference category. Region A/B: these letter codes are used to guarantee anonymity. Other antibiotic use means “antibiotic use (minus florfenicol use)”.

## Data Availability

The data that support the findings of this study are available from the Federal Agency for the Safety of the Food Chain and the Federal Agency for Medicines and Health Products. Restrictions apply to the availability of these data, which were used under license for this study. Data are available from the author(s) with the permission of the Federal Agency for the Safety of the Food Chain and the Federal Agency for Medicines and Health Products.

## Supplementary Materials

The following supporting information can be downloaded at https://www.mdpi.com/article/10.3390/antibiotics13080707/s1. Table S1: Filters used for the literature search in PubMed. References [3,19,20,21,22,23,24,25,26,27,28,29,30,31,32,33,34,35,36,37,38,39,40,41,42,43,44] are cited in the Supplementary Materials.
