# Supplementary material for: Risk Factor Analysis for Occurrence of Linezolid-Resistant Bacteria in the Digestive and Respiratory Tract of Food-Producing Animals in Belgium: A Pilot Study
Source: Antibiotics (Basel). 2024 Jul 29;13(8):707. doi: 10.3390/antibiotics13080707 (PMC11350643; doi:10.3390/antibiotics13080707)
Supplement: Supplementary file 1 [file antibiotics-13-00707-s001.zip › antibiotics-3120815-supplementary.pdf]

Supplementary Material

Table S1. Filters used for the literature search in PubMed.

		**Number of publications**	**Relevant publications**
**Search terms**	Calves, Antimicrobial use/Antibiotic use, Resistance	37	[3,19,20,31-36]
	Calves, Risk factor, Resistance	10	[19-21,27]
	Pigs, Antibiotic use, Resistance	34	[3,29,37]
	Pigs, Risk factor, Resistance	12	[30,38]
	Broiler, Antimicrobial use/Antibiotic use, Resistance	45	[23,39-41]
	Broiler, Risk factor, Resistance	49	[23,40-44]
	Laying hen/Layer hen, Antibiotic use, Resistance	22	None
	Laying hen/Layer hen, Risk factor, Resistance	2	None
**Search field**	Title and abstract		
**Publication date**	10 Years		
**Species**	Other animals		
**Search date**	2022/03/17		
